# Modulation of the microbiota–enteric nervous system axis by environmental factors: from adaptive plasticity to maladaptive neuroenteric remodeling

**DOI:** 10.3389/fnins.2026.1909617

**Published:** 2026-09-16

**Authors:** Gemma Mazzuoli-Weber, Kristin Elfers

**Affiliations:** 1 Institute for Physiology and Cell Biology, University of Veterinary Medicine Hannover, Hannover, Germany; 2 Center for Systems Neuroscience (ZSN), Hannover, Germany

**Keywords:** enteric neurons, environmental exposures, gut-brain axis disorders, microbial metabolites, microbiota-gut-brain axis

## Abstract

The enteric nervous system (ENS) is increasingly recognized as a highly plastic integrative network positioned at the interface between the intestinal microbiota, epithelial barrier, immune system, and gut–brain axis. Beyond its established role in regulating gastrointestinal motility, secretion, and blood flow, the ENS continuously senses and integrates microbial, metabolic, immune, and environmental signals to maintain intestinal homeostasis and coordinate adaptive physiological responses. Recent advances have revealed that microbiota-derived metabolites, including short-chain fatty acids, bile acid derivatives, tryptophan metabolites, and neuroactive compounds, can influence enteric neuronal activity and neuroepithelial communication through multiple direct and indirect pathways. These include signaling involving enteric neurons and glia as well as epithelial, enteroendocrine, immune, metabolic, and barrier-dependent mechanisms. Environmental factors including diet, antibiotic exposure, pollutants, and neuroactive substances can further modulate these interactions by altering microbial composition and metabolism, epithelial integrity, immune signaling, and neuronal responsiveness. In this review, we critically examine the evidence supporting these different routes of microbiota–ENS communication and propose a conceptual framework distinguishing acute modulation and adaptive ENS plasticity from maladaptive neuroenteric remodeling. Whereas adaptive plasticity maintains or restores gastrointestinal homeostasis, sustained or insufficiently reversible functional, neurochemical, cellular, and structural alterations may contribute to persistent neuroenteric dysfunction. We discuss how exposure intensity and duration, developmental timing, reversibility, and host susceptibility may influence this transition and consider its relevance to gastrointestinal, inflammatory, and neurodegenerative disorders. Finally, we critically assess current therapeutic approaches and highlight the limited direct clinical evidence for restoration of ENS function. A deeper mechanistic understanding of microbiota–ENS communication and the transition from adaptive to maladaptive responses may provide new opportunities for precision-targeted therapeutic strategies in gastrointestinal and gut-brain disorders.

## Graphical Abstract

**Figure:**
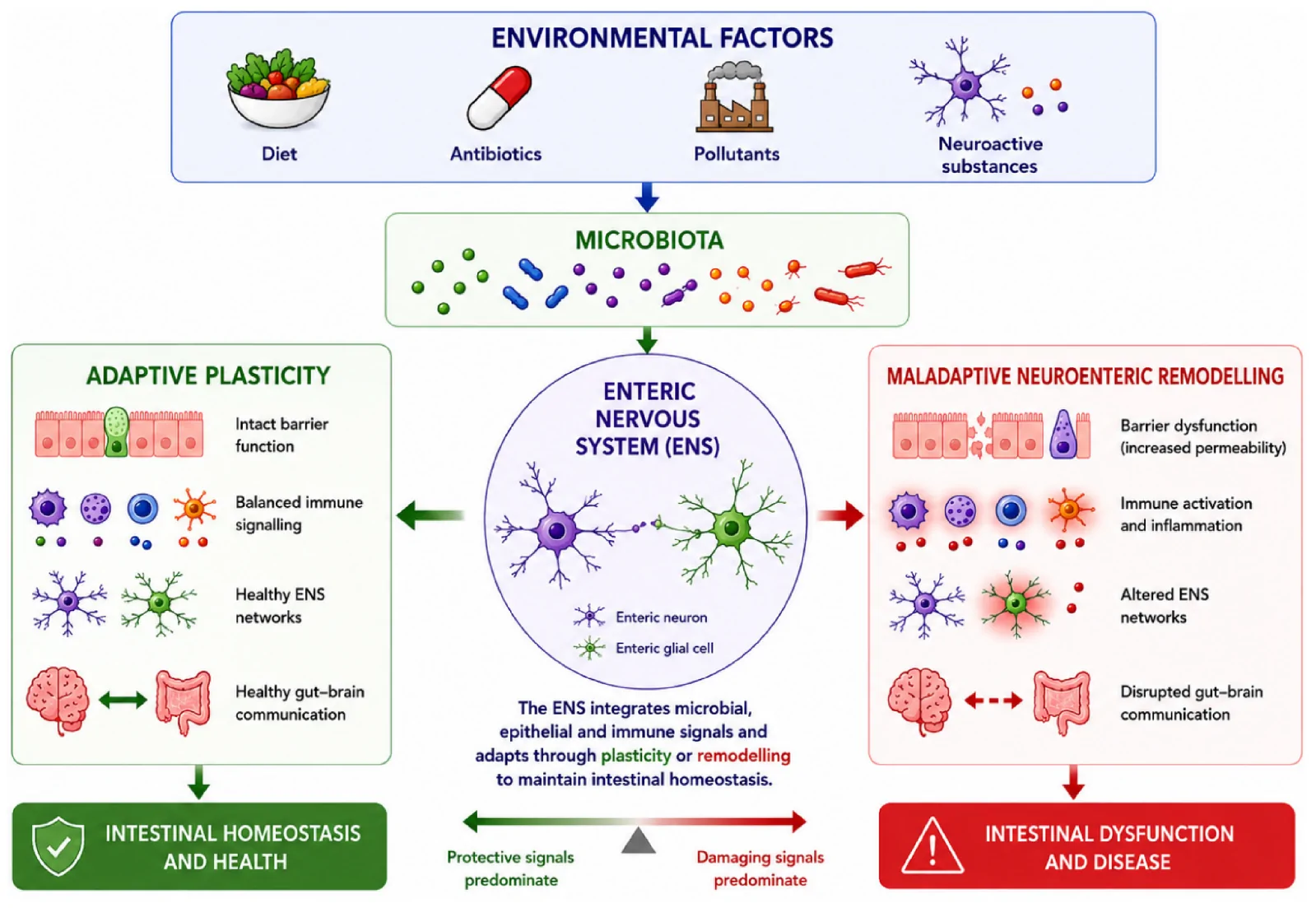
Environmental modulation of enteric nervous system plasticity. Environmental factors, including diet, antibiotics, pollutants, and neuroactive substances, can influence the microbiota–epithelial–immune–ENS axis. The ENS integrates these signals and responds dynamically through adaptive plasticity or maladaptive neuroenteric remodelling. The balance between these outcomes is influenced by the intensity and duration of exposure, host susceptibility, and reversibility. Predominantly protective and reversible responses support intestinal homeostasis and healthy gut–brain communication, whereas persistent damaging signals may promote barrier dysfunction, immune activation, altered ENS networks, disrupted gut–brain signalling, and intestinal disease.

## 1 Introduction

The intestinal microbiota and the enteric nervous system (ENS) form a dynamic interface through which luminal, metabolic, immune, and environmental signals can influence gastrointestinal function and gut–brain communication. Increasing evidence indicates that microbial metabolites and microbiota-dependent host signaling pathways modulate enteric neuronal and glial function, while environmental exposures can alter these interactions by affecting microbial composition, epithelial integrity, immune activity, and neural responsiveness. The ENS is therefore increasingly recognized not simply as an effector of gastrointestinal function, but as a plastic integrative network capable of adapting to changes in the intestinal environment.

Despite rapid progress in this field, several important conceptual and mechanistic gaps remain. Evidence for microbiota–ENS communication ranges from microbial production of candidate neuroactive metabolites and receptor expression within the ENS to functional responses of isolated enteric neurons and *in vivo* alterations in motility or secretion. These different levels of evidence are frequently considered together, making it difficult to distinguish direct effects on enteric neurons or glia from signaling mediated through epithelial, enteroendocrine, immune, metabolic, or barrier-dependent pathways. Moreover, although changes in ENS excitability, neurochemical coding, glial activity, and neuronal structure have been reported following microbial or environmental perturbations, it remains unclear when such responses represent reversible physiological adaptation and when they progress toward persistent and functionally detrimental remodeling.

In this review, we therefore critically examine current evidence for microbiota–ENS communication and its modulation by environmental influences, with particular emphasis on the cellular and molecular routes through which microbial and environmental signals affect enteric neural circuits. We introduce a conceptual framework distinguishing acute modulation and adaptive ENS plasticity from maladaptive neuroenteric remodeling and discuss experimental criteria that may help differentiate these states. Given the breadth of environmental influences, selected exposures are used as mechanistically informative examples rather than as an exhaustive survey of the exposome. Finally, we evaluate the relevance of these processes to gastrointestinal and gut–brain disorders, critically assess the evidence supporting current therapeutic approaches, and identify methodological and translational priorities required to establish causal links between microbiota-associated signals, ENS remodeling, and disease.

## 2 The ENS as a plastic integrative organ at the interface

The gastrointestinal tract is a unique organ system responsible for the digestion and absorption of nutrients while simultaneously being exposed to environmental microbes and pollutants. It also plays a central role in mediating the complex crosstalk among the microbiome, the immune system, and the body’s internal milieu. To coordinate all these functions, the gastrointestinal tract is equipped with an intrinsic nervous system, known as the enteric nervous system (ENS), which regulates motility, secretion, blood flow, and communication with the immune system and the central nervous system (Furness, 2012; Costa and Brookes, 2008; Spencer and Hu, 2020).

The ENS comprises hundreds of millions of neurons organized in the myenteric and submucosal plexuses. This complex neuronal network, embedded within the wall of the entire gastrointestinal tract, independently controls epithelial and motoric gastrointestinal functions and exhibits a high degree of plasticity and integrative capacity.

Functionally, the ENS integrates a wide spectrum of inputs, including luminal metabolites, microbial-derived products, hormones, immune mediators, and environmental signals. Its remarkable neurotransmitter diversity – including acetylcholine, serotonin (5-HT), vasoactive intestinal peptide, substance P, and nitric oxide – enables fine-tuned and context-dependent modulation of intestinal physiology. Importantly, this neurochemical versatility supports bidirectional communication with epithelial, immune, and microbial compartments (Costa et al., 2021).

Growing evidence indicates that the ENS functions as a dynamic sensory network that directly monitors the intestinal milieu while continuously adapting to environmental inputs (Figure 1). *Ex vivo* neuroimaging and electrophysiological studies have shown that enteric neuronal activity is regulated not only by nutrients but also by luminal and fecal constituents, including microbial-derived metabolites such as SCFAs and bile acid derivatives (Boesmans et al., 2013; Wang et al., 2025; Fung et al., 2025; Elfers et al., 2024). In addition, environmental and pharmacological factors have been shown to directly affect enteric neuronal function. Exposure to nano- and micro-polystyrene particles alters intestinal epithelial barrier properties and modulates enteric neuronal activity *in vitro*, highlighting a potential impact of environmental microplastic contamination on gut neurophysiology (Elfers et al., 2025a). Likewise, both acute and prolonged cocaine exposure induce marked changes in enteric neuronal excitability and network activity, demonstrating the susceptibility of ENS signaling to neuroactive substances (Elfers et al., 2023). Beyond acute sensing, enteric neuronal excitability and synaptic transmission exhibit substantial plasticity in response to luminal and pharmacological stimuli. Dietary factors can influence ENS structure and function through mechanisms involving microbial metabolism, immune signaling, and direct neuroactive actions, resulting in alterations in neuronal phenotype, neurotransmitter expression, and circuit organization (Wang et al., 2025). Collectively, these findings underscore the ENS as an adaptive neurobiological interface that integrates dietary, microbial, environmental, and pharmacological cues to regulate gastrointestinal physiology and undergo continual structural and functional remodeling.

**FIGURE 1:**
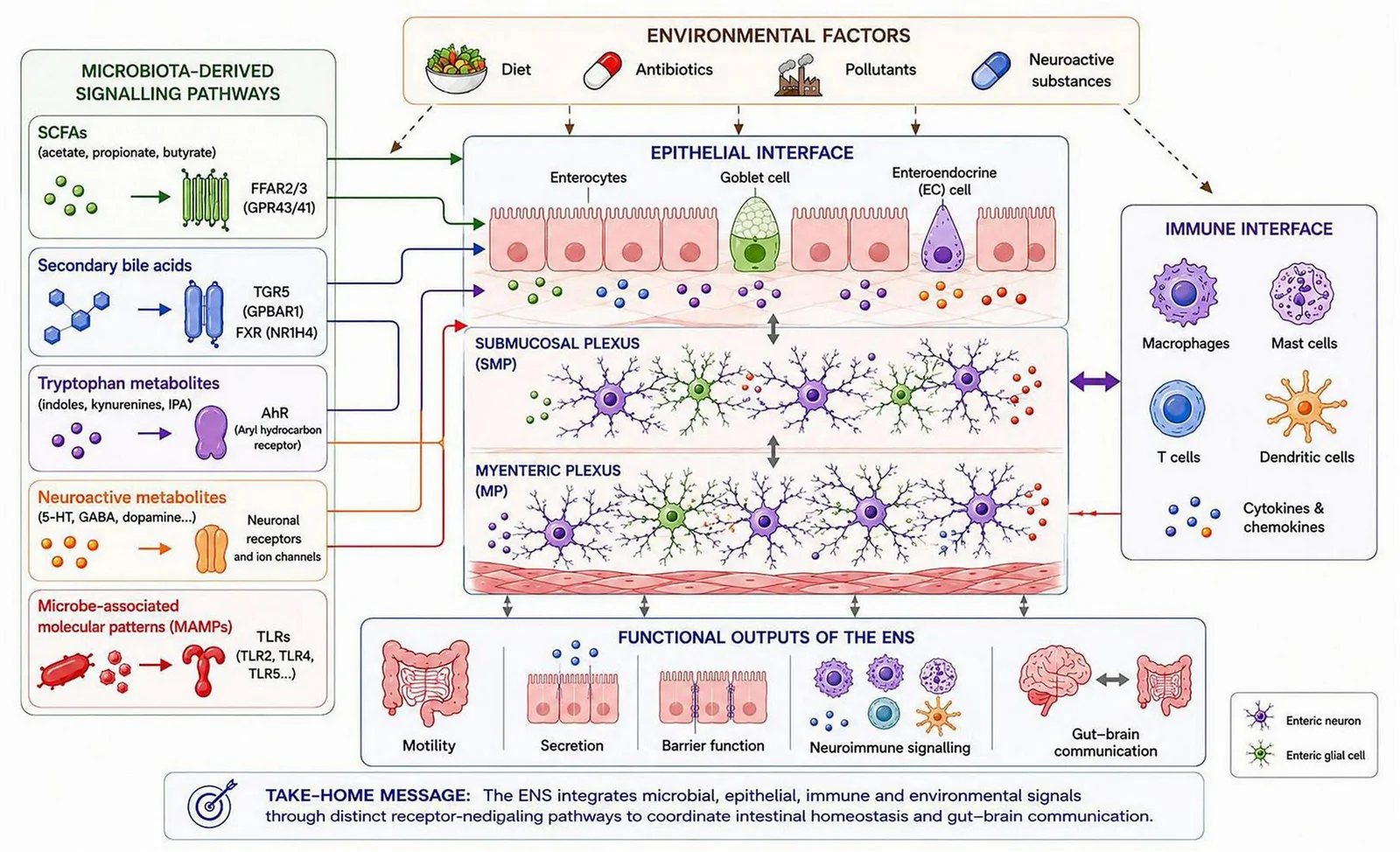
The enteric nervous system as central integrator of microbial, epithelial, immune and environmental signals. The enteric nervous system (ENS) receives and integrates signals originating from the intestinal microbiota, epithelial barrier, immune system, and environmental factors. Microbiota-derived metabolites, including short-chain fatty acids (SCFAs), secondary bile acids, tryptophan metabolites, neuroactive compounds, and microbial-associated molecular patterns (MAMPs), interact with epithelial, immune, neuronal, and glial pathways. Through bidirectional communication with epithelial and immune cells, the ENS coordinates intestinal motility, secretion, barrier integrity, neuroimmune signaling, and gut–brain communication, thereby maintaining intestinal homeostasis. AHR, aryl hydrocarbon receptor; ENS, enteric nervous system; FFAR2, free fatty acid receptor 2; FFAR3, free fatty acid receptor 3; GABA, γ-aminobutyric acid; 5-HT, 5-hydroxytryptamine; SCFAs, short-chain fatty acids.

## 3 Microbial signals shape ENS structure and function

### 3.1 Short-chain fatty acids as key neuromodulatory metabolites

SCFAs, including acetate, propionate, and butyrate, represent central microbiota-derived signaling molecules able to modulate intestinal physiology and ENS functionality (Koh et al., 2016; Soret et al., 2010). Acting primarily via FFA2 and FFA3 receptors expressed on epithelial and neuroepithelial interfaces, including FFAR3/GPR41 expression within enteric neuronal networks (Nøhr et al., 2013), SCFAs regulate epithelial secretion, barrier integrity, and serotonergic and cholinergic signaling pathways (Soret et al., 2010; Ballout et al., 2021; Mishra et al., 2020). Beyond their effects on epithelial homeostasis, SCFAs directly modulate ENS activity and intestinal motility. In particular, butyrate increases the proportion of cholinergic myenteric neurons *in vivo* after resistant starch feeding or intracecal infusion, concomitant with enhanced colonic motility, while direct application of butyrate to cultured myenteric ganglia similarly promotes a cholinergic neuronal phenotype, indicating also direct action on enteric neurons (Soret et al., 2010).

In contrast, propionate appears to exert distinct and partly opposing effects on intestinal physiology by reducing colonic motility while simultaneously stimulating epithelial secretory activity and increasing the abundance of vasoactive intestinal peptide-positive enteric neurons (Yajima et al., 2011; Hurst et al., 2014; De Vadder et al., 2015). Notably, De Vadder et al. demonstrated that microbiota-derived propionate and butyrate activate intestinal gluconeogenesis through gut–brain neural pathways, highlighting the role of the gut–brain axis as a key route for microbial metabolites to directly influence neuronal signaling networks and host physiology (De Vadder et al., 2014).

Collectively, these findings highlight the complex and metabolite-specific roles of SCFAs in coordinating neuroepithelial signaling, intestinal secretion, ENS-dependent motility regulation and shaping enteric neuronal excitability.

### 3.2 Mechanisms of microbiota–ENS communication

Microbiota–ENS communication can occur through multiple routes that differ substantially in their degree of directness. Given the anatomical separation between the intestinal lumen and most enteric neuronal and glial networks, the presence of a microbial metabolite in the lumen or an association between microbiota composition and ENS function does not by itself demonstrate direct neuronal signaling. Here, we distinguish direct ENS effects, defined as responses of enteric neurons or glia to a microbial product demonstrated in isolated cells, ganglia, or neural preparations, from indirect communication mediated through epithelial or enteroendocrine cells, immune mediators, metabolite absorption, barrier-dependent mechanisms, or other intermediary cell types. Evidence for microbial production of a candidate mediator, expression of its receptor within the ENS, and *in vivo* changes in motility or secretion represent complementary but distinct levels of evidence and should not be interpreted as equivalent demonstrations of direct microbiota–ENS signaling.

Emerging evidence indicates that microbiota–ENS communication extends far beyond SCFAs and involves a diverse spectrum of microbial metabolites capable of influencing epithelial, immune, and neuronal signaling pathways. Rather than acting through isolated mechanisms, many of these metabolites appear to converge on common regulatory processes controlling enteric neuronal excitability, neuroepithelial communication, and intestinal motility.

Secondary bile acids generated by intestinal bacteria can modulate epithelial signaling cascades and intestinal transit, while microbiota-derived tryptophan metabolites regulate mucosal immune responses and 5-HT-associated regulatory pathways (Ridlon et al., 2006; Zelante et al., 2013; Yano et al., 2015). In parallel, several intestinal bacterial species produce neuroactive compounds including GABA, catecholamine-like molecules, and 5-HT precursors, thereby providing additional mechanisms through which microbial activity may influence ENS function and communication (Barrett et al., 2012; Asano et al., 2012; Ren et al., 2017).

Although many of these mechanisms remain incompletely resolved, accumulating evidence suggests that microbiota-derived metabolites can influence neuronal responsiveness both directly and indirectly through coordinated modulation of epithelial secretion, immune signaling, and neuroepithelial communication.

### 3.3 Pattern recognition receptors in the ENS

A crucial yet underexplored mechanism of direct microbiota–ENS interaction involves pattern recognition receptors (PRRs) expressed on enteric neurons and enteric glial cells. Receptors such as Toll-like receptors (TLRs) and other PRRs enable the ENS to detect microbial-associated molecular patterns (MAMPs), linking microbial sensing to neuronal plasticity and neuroimmune modulation (Brun et al., 2013; Barajon et al., 2009). In addition to their established roles in innate immune signaling, PRR-dependent pathways within the ENS have been implicated in the regulation of inflammatory mediator release, enteric neuronal responsiveness, and intestinal motility, suggesting that microbial sensing mechanisms directly contribute to the functional modulation of enteric neural circuits (Coquenlorge et al., 2014; Faggin et al., 2025). Emerging evidence identifies enteric glial cells as central mediators of microbiota–host communication, linking microbial signals with epithelial barrier regulation, immune responses, and ENS function. Enteric glia also express multiple TLRs, including TLR3, TLR4, and TLR7, supporting their ability to directly sense microbial-associated signals (Barajon et al., 2009). Furthermore, TLR2-dependent signaling is required for maintaining ENS integrity and limiting intestinal inflammation, underscoring the importance of glial innate immune sensing in gastrointestinal homeostasis (Brun et al., 2013). Together, these observations position enteric glia as key regulators of neuroimmune crosstalk and intestinal tissue homeostasis (Kabouridis et al., 2015). This dual sensing capacity suggests that the ENS integrates both metabolic and immunological microbial signals, reinforcing its role as a central hub in microbiota–host communication. However, the precise molecular and cellular mechanisms by which microbial sensing pathways shape ENS development, long-term plasticity, and neuroimmune remodeling under physiological and pathological conditions remain insufficiently understood and require further mechanistic investigation.

### 3.4 Functional evidence for neuroactive effects of luminal microbial factors

Application of fecal supernatants to *ex vivo* myenteric preparations can directly alter enteric neuronal activity, demonstrating that soluble luminal factors possess neuroactive properties (Elfers et al., 2024). However, such experiments do not establish whether the active compounds are of microbial or host origin, nor do they identify the neuronal receptors or signaling pathways involved. They therefore provide evidence for a direct neuroactive effect of soluble fecal factors on the ENS, but not, by themselves, for direct microbiota–neuron signaling. Complementary studies using germ-free and microbiota-manipulated models demonstrate that the presence and composition of the intestinal microbiota influence ENS maturation, neuronal excitability, neurochemical coding, and intestinal function, although these effects may involve epithelial, immune, metabolic, or other intermediary pathways (Obata et al., 2020; De Vadder et al., 2018). Together, these different levels of evidence support the intestinal microbiota as an important regulator of ENS structure and function, while highlighting that the cellular routes and molecular mediators linking microbial activity to enteric neural circuits remain incompletely resolved. The different levels of evidence supporting microbiota- and environment-associated modulation of ENS function are summarized in Table 1.

**TABLE 1:** Evidence for microbiota- and environment-associated modulation of ENS function.

Signal / exposure	Proposed mediator	Cellular target	Receptor/ pathway	Experimental model	Effect	Direct vs. indirect evidence	Human / clinical evidence	Principal limitation
Butyrate / SCFAs (Soret et al., 2010)	Butyrate	Myenteric neurons	Not fully resolved	Rat *in vivo*; primary ENS cultures	Increased cholinergic neuronal phenotype; enhanced colonic motility	Direct ENS	No direct human ENS evidence	Direct neuronal receptor mechanism not established
Microbiota-derived metabolites / 5-HT pathway (Yano et al., 2015)	Microbial metabolites stimulating host 5-HT production	Enterochromaffin cells	Host serotonergic signaling	GF mice; colonization; EC cultures	Increased colonic 5-HT; altered GI motility	Indirect via EC cells	Human-derived bacterial isolates included; no direct human ENS evidence	Demonstrates microbial regulation of host 5-HT, not direct ENS activation
Microbiota colonization (De Vadder et al., 2018)	Host 5-HT	ENS neuronal precursors / neurons	5-HT4 receptor	GF and conventionalised mice	ENS maturation, altered neuroanatomy and intestinal transit	Indirect/mechanistic	No	Mediator is host-derived; direct microbial–neuron contact not demonstrated
Microbiota-dependent luminal signals (Obata et al., 2020)	Microbiota-dependent AHR ligands	Enteric neurons	AHR–CYP1A1 signaling	Antibiotic-treated and genetically modified mice; neuronal transcriptomics	Neuronal transcriptional programming; regulation of peristalsis	ENS-specific mechanistic evidence	No	Individual microbial ligand(s) and route from lumen to neuron not fully defined
Antibiotic-induced microbiota depletion (Vicentini et al., 2021)	Altered microbiota / microbial signals	Enteric neurons and glia; epithelial compartment	Multiple / incompletely defined	Antibiotic-treated mice with spontaneous microbiota recovery	Altered neuronal/glial populations, permeability and transit; recovery after microbiota restoration	Indirect	No	Broad microbiota perturbation prevents attribution to a specific mediator
Fecal supernatants (Elfers et al., 2024)	Soluble fecal factors; SCFAs analysed as candidates	Cultured myenteric neurons	Unknown	Canine fecal supernatant samples applied to cultured myenteric neurons	Direct neuronal activation and altered burst activity	Direct functional ENS effect of fecal supernatant	Naturally occurring canine disease; no human data	Active mediator, microbial vs. host origin, and neuronal receptor unknown
Fecal supernatants (Ridz̆al et al., 2026)	Soluble fecal factors, incl, proteases	Submucous neurons in wholemount preparations	PAR1/PAR2	Fecal supernatant samples from human IBS patients applied to submucosal wholemount preparations from the guinea pig	Direct neuronal activation and altered burst frequency	Direct functional ENS effect of fecal supernatant	Human disease with high prevalence	Complexity of fecal supernatants; cross-species model
Nano-/micro-polystyrene (Elfers et al., 2025a)	Polystyrene particles and/or secondary epithelial signals	Intestinal epithelium; enteric neurons	Unknown	*In vitro* intestinal epithelial and ENS preparations	Altered epithelial transport and enteric neuronal activity	Direct experimental exposure; pathway unresolved	No	*In vitro* exposure; relevance of concentrations and *in vivo* exposure remains uncertain
Cocaine (Elfers et al., 2023)	Cocaine	Enteric neurons; epithelial secretory pathways; smooth muscle	Nicotinic and serotonergic responses affected	Guinea pig primary neurons, neuroimaging, Ussing chamber, organ bath	Acute suppression of neuronal responses and neuronally mediated secretion; altered GI functions	Direct pharmacological ENS evidence; not microbiota-mediated	GI symptoms associated with cocaine use, but no direct human ENS data in study	Experimental exposure; relationship to microbiota–ENS signaling not established

Direct ENS evidence refers to experiments in which a defined compound or complex biological preparation was applied directly to isolated enteric neurons, ganglia, or ENS-containing preparations and neuronal responses were measured. This classification does not necessarily imply direct access of the respective signal to enteric neurons *in vivo*. Indirect evidence refers to effects requiring or potentially involving epithelial, enteroendocrine, immune, metabolic, barrier-dependent, or other intermediary pathways. 5-HT, serotonin; AHR, aryl hydrocarbon receptor; ENS, enteric nervous system; FFAR2, free fatty acid receptor 2; FFAR3, free fatty acid receptor 3; GF, germ-free; GI, gastrointestinal; SCFAs, short-chain fatty acids.

### 3.5 Environmental factors as modulators of the microbiota–ENS axis

Environmental influences on the microbiota–ENS axis encompass a broad and heterogeneous range of exposures. Conceptually, these can be grouped into nutritional factors, including dietary patterns and food additives; pharmacological exposures, including antibiotics and other medications; psychosocial and behavioral factors, such as stress, physical activity, smoking, and alcohol consumption; biological exposures, including intestinal infections; environmental contaminants, including micro- and nanoplastics, pesticides, metals, and air pollutants; and early-life exposures occurring during critical periods of microbiota and ENS development. These factors differ substantially in their routes of exposure and primary biological targets and should therefore not be considered a mechanistically uniform group. Rather, they may converge at different levels of the microbiota–ENS interface by modifying microbial community structure and metabolism, epithelial and immune signaling, or neuronal and glial function.

Given the breadth of this field, the following sections do not aim to provide an exhaustive catalog of environmental exposures. Instead, we focus on selected examples for which experimental evidence allows links between environmental exposure, microbiota-associated changes, and ENS function or plasticity to be considered. Diet and antibiotics illustrate physiological and pharmacological perturbations of the intestinal microbial ecosystem, whereas micro- and nanoplastics and neuroactive substances provide examples of emerging environmental and lifestyle-associated exposures with evidence of direct or indirect effects on enteric neural signaling.

#### 3.5.1 Diet, microbiota depletion and ENS plasticity

Dietary intake and antibiotic exposure represent major environmental determinants of gut microbiota composition and microbial metabolite availability. Through their effects on microbial community structure, these factors can profoundly influence epithelial homeostasis, neuroepithelial communication, and ENS function. In particular, high-fiber diets promote the production of microbiota-derived SCFAs, which modulate intestinal secretion, motility, and enteric neuronal signaling through epithelial and neuroepithelial pathways (De Vadder et al., 2014; Ballout et al., 2025). Conversely, antibiotic-induced dysbiosis disrupts microbial metabolite production and has been associated with altered serotonergic signaling, impaired intestinal motility, and disturbed ENS activity (Ge et al., 2017; Vicentini et al., 2021).

Importantly, germ-free (GF) and microbiota-depletion models have provided compelling evidence that microbial colonization is required for normal ENS maturation and functional organization. GF animals display altered intestinal motility, impaired enteric neuronal excitability, and changes in enteric neurochemical coding, demonstrating a fundamental role of the microbiota in shaping ENS structure and function (Husebye et al., 2001; De Vadder et al., 2018). Moreover, recolonization studies indicate that several microbiota-dependent ENS alterations are at least partially reversible. Restoration of the intestinal microbiota following GF conditions or antibiotic-induced depletion can normalize enteric neuronal density, neurochemical coding, and intestinal motility, highlighting the remarkable plasticity of the microbiota–ENS axis in adulthood (De Vadder et al., 2018; Vicentini et al., 2021).

In addition to acute functional modulation, environmental perturbations of the microbiota may exert long-term effects on neurodevelopmental and gut–brain signaling pathways. Early-life microbiota depletion induced by broad-spectrum antibiotic treatment has been associated with persistent alterations in neural signaling and behavior, emphasizing the importance of microbial colonization during critical developmental windows (Desbonnet et al., 2015). Collectively, these findings support the concept that environmental factors dynamically shape ENS structure and function through microbiota-dependent mechanisms and that chronic perturbations may promote maladaptive neuroenteric remodeling.

#### 3.5.2 Environmental pollutants and neuroactive exposures as modulators of ENS function

Environmental pollutants and neuroactive exposures can influence intestinal homeostasis through multiple and partly interconnected mechanisms involving the microbiota, epithelial barrier, immune system, and ENS. Micro- and nanoplastics (MNPs) have received increasing attention because experimental exposure has been associated with alterations in microbial composition and metabolism, epithelial barrier function, and intestinal inflammatory responses. However, reported effects vary considerably depending on particle size, polymer composition, concentration, exposure duration, and experimental model, and some studies have reported limited particle uptake or overt intestinal toxicity (Stock et al., 2019; Jin et al., 2018; Zhang et al., 2024). Mechanistically, polystyrene microplastic exposure has been associated with reduced mucus secretion, intestinal barrier dysfunction, microbiota dysbiosis, and alterations in metabolic pathways, including bile acid and amino acid metabolism (Jin et al., 2018). More recent experimental evidence links polystyrene microplastic-induced epithelial barrier dysfunction to oxidative stress and activation of ROS-dependent NF-κB/NLRP3 signaling. Downstream inflammatory signaling involving IL-1β and MLCK was associated with reduced expression of tight-junction proteins, including ZO-1, occludin, and claudin-1 (Zeng et al., 2024). These findings provide a potential mechanistic sequence whereby particulate exposure induces epithelial and inflammatory changes that could indirectly modify the microenvironment surrounding enteric neurons and glia. However, a causal link between these molecular pathways and altered ENS function has not yet been demonstrated.

In addition to such indirect mechanisms, experimental evidence indicates that plastic particles can alter enteric neuronal function. Exposure of primary cultured myenteric neurons to nano- and micro-polystyrene particles modified neuronal responses to nicotinic stimulation following 24 h exposure, while experiments in native intestinal tissue demonstrated particle size-dependent effects on epithelial electrophysiological properties (Elfers et al., 2025a). Notably, these functional changes occurred without evidence of overt tissue damage, suggesting that neuroepithelial dysfunction may precede structural injury. Nevertheless, the molecular sensors and signaling pathways underlying the neuronal effects remain unknown, and it has not yet been established whether particle exposure affects enteric neurons directly *in vivo* or through epithelial, immune, or other intermediary mechanisms.

Neuroactive substances may affect gut physiology through a mechanistically distinct route by interfering with neurotransmission within enteric neural circuits. Cocaine provides an experimental example of such modulation. In primary cultures of guinea-pig myenteric neurons, acute cocaine exposure attenuated neuronal responses to nicotinic and serotonergic stimulation, demonstrating an acute effect on enteric neuronal responsiveness (Elfers et al., 2023). In parallel, cocaine reversibly reduced neuronally mediated mucosal secretion in intestinal tissue preparations, linking altered neural signaling to a functional epithelial output. In contrast, following 24 h exposure, neuronal responses to nicotinic and serotonergic stimulation were no longer significantly different from controls, suggesting adaptation or tolerance rather than persistent neuronal dysfunction. Although cocaine is known to interfere with monoaminergic neurotransmission, the specific molecular targets responsible for the observed ENS effects were not identified in these experiments (Elfers et al., 2023).

These findings highlight the remarkable plasticity of the ENS and demonstrate its susceptibility to environmental agents capable of directly modulating neurotransmission and circuit function.

Collectively, these studies illustrate that environmental pollutants and neuroactive substances can affect gut physiology through distinct but convergent mechanisms. Particulate pollutants may act upstream through microbiota alterations, oxidative stress, epithelial barrier dysfunction, and inflammatory signaling, with additional experimental evidence for altered enteric neuronal responsiveness. Neuroactive compounds can more directly modify enteric neurotransmission and downstream gastrointestinal functions. Importantly, however, functional effects on enteric neurons should not be equated with a defined molecular mechanism or with direct neuronal exposure *in vivo*. Establishing how exposure intensity and duration, epithelial and immune responses, and direct neural effects interact will be essential to determine whether environmental exposures induce transient ENS modulation, reversible adaptation, or persistent maladaptive neuroenteric remodeling.

For several other environmental and lifestyle factors, including psychosocial stress, physical activity, smoking, alcohol consumption, non-antibiotic medications, infections, pesticides, metals, and air pollutants, effects on the intestinal microbiota, epithelial barrier, or immune system have been reported, whereas direct evidence for consequent ENS functional or structural remodeling remains comparatively limited. Early-life exposures may be particularly relevant because microbial colonization and ENS maturation occur concurrently, potentially creating critical windows of susceptibility. Determining whether these diverse exposures induce reversible ENS adaptation or persistent maladaptive remodeling represents an important direction for future research.

### 3.6 From adaptive to maladaptive neuroenteric remodeling

Physiological modulation of the microbiota-ENS axis is essential for maintaining intestinal homeostasis and coordinated gut-brain communication. Under physiological conditions, enteric neuronal networks continuously integrate microbial metabolites, dietary components, immune mediators, and luminal signals to dynamically regulate intestinal motility, secretion, epithelial barrier function, and neuroimmune interactions. This intrinsic plasticity enables the ENS to adapt to changing environmental and microbial conditions while preserving intestinal function and homeostatic signaling.

For this review, we distinguish acute modulation, adaptive plasticity, and maladaptive neuroenteric remodeling as related but conceptually distinct processes. Acute modulation refers to rapid, stimulus-dependent changes in neuronal excitability, synaptic transmission, or epithelial–neural communication that resolve when the stimulus is removed and do not necessarily imply plasticity. Adaptive plasticity encompasses functional, neurochemical, or cellular changes that allow the ENS to accommodate altered luminal, microbial, immune, or environmental conditions while maintaining or restoring gastrointestinal homeostasis. Such responses may include reversible changes in neuronal excitability, neurotransmitter expression, synaptic efficacy, or glial activity.

In contrast, we define maladaptive neuroenteric remodeling as sustained functional, neurochemical, cellular, or structural alterations of enteric neural circuits that impair, rather than preserve, physiological function. These changes may include persistent alterations in neuronal excitability and network behavior, molecular and neurochemical changes in neuronal or glial signaling pathways, neurochemical recoding, sustained enteric glial or neuroimmune activation, altered neuronal connectivity, and, in more severe conditions, enteric neuropathy or neuronal loss. Such molecular changes may involve altered neurotransmitter or receptor expression and activation of inflammatory or cell-death signaling pathways, but no molecular marker has yet been established that specifically defines maladaptive ENS remodeling (Neunlist et al., 2003; Gulbransen et al., 2012). Importantly, neuronal loss is therefore considered a possible consequence of severe maladaptive remodeling rather than a prerequisite for its definition. Persistence after withdrawal of the initiating stimulus provides particularly strong evidence for maladaptive remodeling, but should not be regarded as an absolute requirement, since chronic exposures may continuously drive dysfunctional neural states. Moreover, neuronal loss does not necessarily translate into impaired function, as surviving neurons may retain functional capacity and compensate for the loss of neuronal cells.

The transition between adaptive and maladaptive responses is unlikely to represent a discrete threshold. Instead, we propose that it is determined by the interaction between exposure intensity and duration, developmental timing, reversibility, and host susceptibility (Figure 2). Accordingly, the same stimulus may induce reversible adaptation under one set of conditions but persistent dysfunction under another. This framework generates experimentally testable predictions: adaptive responses should return toward baseline following removal of the initiating stimulus, whereas maladaptive remodeling should show delayed or incomplete recovery and remain associated with impaired physiological function. Longitudinal assessment of neuronal excitability and network activity, molecular and neurochemical phenotyping, receptor and signaling-pathway expression, enteric glial activation, neuronal density and morphology, together with functional measures such as motility, secretion, barrier function, and visceral sensitivity, could therefore provide operational endpoints for identifying the transition from adaptation to maladaptation.

**FIGURE 2:**
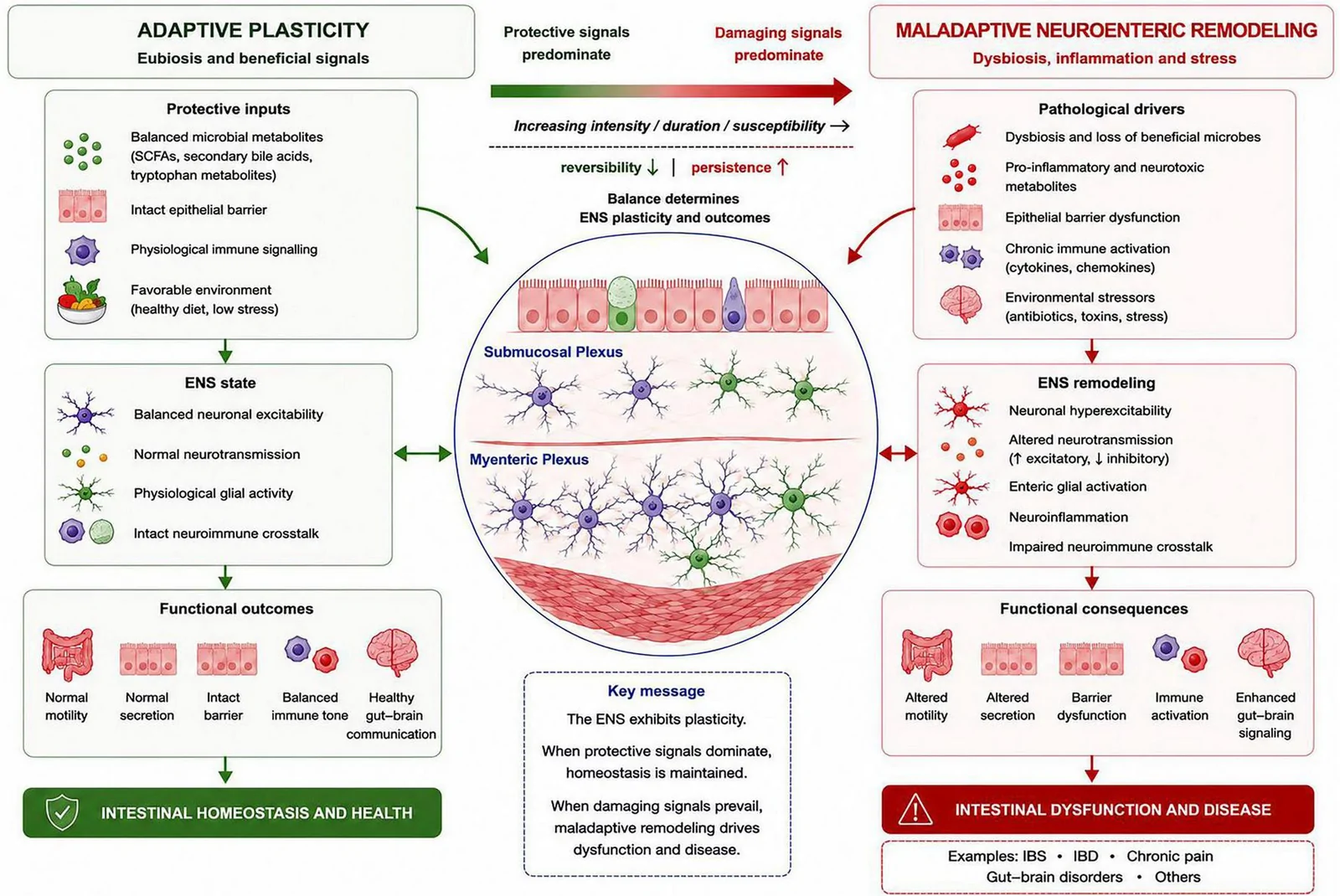
From adaptative plasticity to maladaptive neuroenteric remodeling. The ENS exhibits substantial functional plasticity in response to microbial, immune, epithelial, and environmental cues. Under physiological conditions, balanced microbial metabolites, an intact epithelial barrier, and regulated immune signaling promote adaptive ENS plasticity, resulting in normal neuronal excitability, physiological glial activity, and maintenance of intestinal homeostasis. In contrast, dysbiosis, epithelial barrier dysfunction, chronic immune activation, and adverse environmental exposures can drive maladaptive neuroenteric remodeling characterized by altered neurotransmission, enteric glial activation, neuroinflammation, and disrupted neuroimmune communication. The transition between these states is conceptualized as a continuum influenced by the intensity and duration of the initiating stimulus and by host susceptibility, with decreasing reversibility and increasing persistence favoring maladaptive remodeling. These changes contribute to impaired intestinal function, altered gut-brain signaling, and the development of gastrointestinal and extra-intestinal disorders. CNS, central nervous system; ENS, enteric nervous system; IBD, inflammatory bowel disease; IBS, irritable bowel syndrome; SCFAs, short-chain fatty acids.

Experimental evidence supports several components of this proposed continuum. Disturbances in microbiota-dependent signaling can alter enteric neuronal excitability, serotonergic signaling pathways, intestinal motility, epithelial barrier integrity, and neuroepithelial communication (Ge et al., 2017; De Vadder et al., 2018; Vicentini et al., 2021). Importantly, microbiota depletion and recolonization studies demonstrate that at least some of these alterations are reversible, supporting their interpretation as adaptive or plastic responses rather than persistent remodeling (De Vadder et al., 2018; Vicentini et al., 2021). In contrast, sustained intestinal inflammation can induce more persistent functional and structural changes within enteric neural circuits. Alterations in neuronal excitability, neurochemical coding, neuronal density, and ENS network organization have been observed in experimental colitis models and human IBD tissue (Poli et al., 2001; Neunlist et al., 2003; Margolis et al., 2011). Together, these studies illustrate that the nature, duration, and reversibility of ENS alterations vary considerably depending on the initiating stimulus and experimental context.

The functional consequences of such neuroenteric alterations may include disturbed intestinal motility, altered sensory processing, impaired epithelial regulation, and abnormal neuronal activation. In functional gastrointestinal disorders, for example, mucosal mediators from patients with IBS can activate enteric and sensory neural pathways, and mast cell–nerve interactions correlate with abdominal pain and visceral sensitivity (Barbara et al., 2004; Buhner et al., 2014). In inflammatory bowel disease, changes in enteric neuronal phenotype and neuronal integrity further indicate that chronic inflammation can affect both the functional and structural organization of the ENS (Neunlist et al., 2003; Margolis et al., 2011). More recently, Ridzal et al. (Ridz̆al et al., 2026) demonstrated that faecal supernatants from patients with IBS can directly activate enteric neurons in a subtype-dependent manner, with IBS-D-associated neuronal activation linked to increased protease activity and PAR-1 signalling, further supporting a direct functional interface between luminal mediators and the ENS.

Collectively, these findings support a model in which adaptive and maladaptive ENS responses are distinguished not by a single molecular marker, but by the combination of persistence, reversibility, structural involvement, and functional consequences. The ENS is therefore not merely a passive target of microbial and inflammatory alterations, but a dynamic integrative system whose initially adaptive responses may, under sustained or sufficiently intense perturbation, progress toward persistent neuroenteric dysfunction. The conceptual distinction between adaptive and maladaptive neuroenteric responses may also be relevant beyond primary gastrointestinal disorders. The strongest mechanistic evidence currently exists for Parkinson’s disease. Experimental studies have demonstrated that pathological α-synuclein introduced into the gastrointestinal wall can propagate from the enteric nervous system to anatomically connected brain regions via the vagus nerve, with subsequent dopaminergic neurodegeneration and motor and non-motor abnormalities. Importantly, truncal vagotomy prevents this gut-to-brain propagation and associated neurodegenerative phenotypes, supporting a functional role of vagal pathways in the transmission of pathological α-synuclein (Uemura et al., 2018; Kim et al., 2019). However, propagation beyond the dorsal motor nucleus is not observed consistently across experimental models, indicating that additional molecular, cellular, or host-specific factors are likely required for progressive neurodegeneration. Microbiota-dependent mechanisms may further modulate these processes. In α-synuclein-overexpressing mice, the gut microbiota has been shown to influence motor deficits and central neuroinflammation, providing direct evidence that microbial signals can modify disease-associated CNS phenotypes (Sampson et al., 2016). Nevertheless, whether such microbiota-dependent effects are mediated through persistent ENS remodeling, vagal signaling, circulating metabolites, immune pathways, or a combination of these mechanisms remains unresolved.

Evidence for ENS involvement is also emerging in Alzheimer’s disease. In 5xFAD mice, amyloid-β accumulates in enteric neurons and is associated with low-grade intestinal inflammation and altered gastrointestinal transit, although overall ENS organization appears largely preserved (Tasnády et al., 2025). These findings indicate that enteric pathology and gastrointestinal dysfunction can accompany central amyloid pathology without necessarily implying overt enteric neurodegeneration. Collectively, the available evidence supports the possibility that maladaptive neuroenteric processes participate in neurodegenerative disease, but longitudinal studies combining microbiome and metabolomic profiling with direct functional and structural assessment of the ENS are required to determine whether these changes are causal contributors, secondary consequences, or parallel manifestations of systemic disease.

### 3.7 Idiopathic epilepsy in dogs as a translational model

Naturally occurring idiopathic epilepsy (IE) in dogs has recently emerged as a promising translational model within the microbiota–gut–brain axis framework. Experimental and clinical studies have reported alterations in microbial composition and microbial metabolites, particularly in SCFA profiles, in dogs with IE (Watanangura et al., 2022), suggesting that dysbiosis-associated changes in gut–brain signaling may contribute to disease-associated neurophysiological alterations. Supporting this concept, fecal supernatants derived from dogs with IE have been shown to directly activate enteric neurons *ex vivo*, interestingly, to different extents depending on the treatment history and drug responsiveness. This provides functional evidence that microbiota-associated luminal factors, also influenced by drug intervention, can modulate ENS activity in this disease context (Elfers et al., 2024).

More broadly, accumulating evidence indicates that microbial metabolites may influence not only enteric neuronal function but also neuroimmune processes within the central nervous system. For example, Churchward et al. (2023) demonstrated that alterations in SCFA and related carboxylic acid profiles following fecal microbiota transplantation (FMT) collectively attenuated microglial inflammatory responses, highlighting the importance of microbial metabolite networks as mediators of microbiota-dependent gut-brain communication. These observations support the concept that therapeutic modulation of the gut microbiota may affect both enteric and central neural pathways through metabolite-driven mechanisms.

Although the precise contribution of the ENS to canine epilepsy remains largely unexplored, the close anatomical and physiological similarities between the canine and human gastrointestinal systems provide unique opportunities to investigate microbiota-dependent neuroenteric mechanisms in a clinically relevant setting. In contrast to highly controlled rodent models, canine epilepsy represents a naturally occurring disease context that may facilitate translational investigation of microbiota–gut–brain interactions, ENS-associated signaling pathways, and potential microbiota-targeted therapeutic interventions, with fecal microbiota transplantation representing one promising approach.

### 3.8 Translational challenges and therapeutic targeting

#### 3.8.1 Current approaches

Current therapeutic strategies targeting the microbiota–gut–brain axis remain largely indirect and only partially address ENS-specific mechanisms. Existing approaches primarily aim to modulate microbial composition, attenuate intestinal inflammation, or influence central and peripheral neural signaling pathways. These interventions include probiotics and dietary microbiota-modulating strategies as well as FMT as mentioned in the previous section, vagus nerve stimulation, anti-inflammatory therapies, as well as pharmacological neuromodulators targeting serotonergic, cholinergic, or gut–brain signaling pathways.

Importantly, these therapeutic approaches differ substantially in their proximity to the ENS and in the strength of evidence supporting ENS-mediated effects. Microbiota-targeted interventions, including probiotics, dietary strategies, and FMT, primarily act upstream by modifying microbial composition or metabolite availability, whereas anti-inflammatory therapies may influence the ENS indirectly by reducing immune-mediated neuronal and glial alterations. Vagus nerve stimulation modulates neuroimmune pathways but does not directly target intrinsic enteric neural circuits. In contrast, pharmacological agents acting on receptors or signaling pathways expressed by enteric neurons or glia may provide more direct ENS modulation. However, for most currently available interventions, improvements in gastrointestinal or systemic outcomes cannot yet be attributed specifically to restoration of ENS function or reversal of maladaptive neuroenteric remodeling. Current therapeutic approaches and the strength of evidence supporting their involvement in ENS modulation are summarized in Table 2.

**TABLE 2:** Therapeutic approaches relevant to the microbiota–gut–brain axis and evidence for ENS involvement.

Intervention	Primary target / proposed mechanism	Study design / model	Sample size / model	Main outcome	ENS evidence	Evidence level	Principal limitation
Bifidobacterium infantis 35624 (O’Mahony et al., 2005)	Microbiota modulation; immunomodulatory effects	Randomized, placebo-controlled clinical trial in IBS	77 patients randomized to *L. salivarius*, *B. infantis* or placebo for 8 weeks	*B. infantis* improved several IBS symptoms and normalized the IL-10/IL-12 ratio	No direct ENS measurement	Human clinical, indirect ENS relevance	Clinical and immune endpoints only; mechanism cannot be attributed specifically to ENS modulation
Microbiota colonization / restoration (De Vadder et al., 2018)	Microbiota-dependent enteric serotonin signaling	Germ-free and conventionalized mouse experiments	GF and colonized mice; multiple experimental cohorts	Colonization promoted maturation of the adult ENS and normalized microbiota-dependent ENS alterations	Mechanistic ENS involvement	Preclinical mechanistic	Experimental model rather than therapeutic trial; direct microbial–neuronal route not established
FMT-associated metabolite changes (Churchward et al., 2023)	SCFAs and related carboxylic acids	Human FMT-associated metabolomics combined with primary microglial assays	Human samples plus 7 independent primary microglial cultures for cellular experiments	FMT-associated metabolite changes collectively modulated microglial inflammatory responses	No direct ENS evidence	Translational mechanistic	CNS immune endpoint rather than ENS endpoint; effects of individual metabolites difficult to separate
Implanted VNS in Crohn’s disease (Bonaz et al., 2016)	Vagal neuroimmune / inflammatory reflex pathways	Prospective pilot study	7 patients with active Crohn’s disease	Five of seven progressed toward clinical/endoscopic remission; digestive pain decreased; vagal tone improved	No direct intrinsic ENS assessment	Small human pilot study	Very small, uncontrolled cohort; two patients discontinued because of worsening disease
Implanted VNS in Crohn’s disease (Sinniger et al., 2020)	Vagal neuroimmune modulation	12-month prospective pilot study	9 enrolled; detailed 12-month analyses available for 4 patients	Improvements in clinical/endoscopic indices and changes in inflammatory and autonomic measures	No direct ENS assessment	Small longitudinal clinical pilot	Extremely small evaluable cohort and no control group
Implanted VNS in treatment-refractory Crohn’s disease (D’Haens et al., 2023)	Neuroimmune modulation via vagus nerve	Prospective, open-label, multicentre clinical trial	17 implanted; 16 treated for 16 weeks, followed by maintenance	Significant decrease in Crohn’s disease activity index and fecal calprotectin; reduced inflammatory cytokines; endoscopic improvement in a subset	Indirect ENS relevance	Human clinical, open-label	No control group; intrinsic ENS function/remodeling not measured; one serious device-related infection
P2X7R–pannexin-1 pathway inhibition (Gulbransen et al., 2012)	Neuronal P2X7 receptor–Panx1–ASC–caspase signaling	Experimental colitis models	Multiple mouse/genetic/ pharmacological experimental groups	Blocking P2X7R, Panx1, ASC or caspases prevented inflammation-induced enteric neuronal death; Panx1 inhibition preserved colonic motor function	Direct ENS-targeted mechanistic evidence	Preclinical mechanistic	Not a therapeutic clinical study; mainly prevention of inflammation-associated enteric neuronal loss
Fermented milk product with probiotics (Tillisch et al., 2013)	Microbiota-associated gut–brain signaling	Randomized controlled intervention in healthy women; fMRI before/after 4 weeks	*n* = 36 (FMPP *n* = 12; control product *n* = 11; no intervention *n* = 13)	Altered activity in brain networks involved in emotional, viscerosensory and somatosensory processing	No direct ENS evidence; CNS neuronal endpoint	Human mechanistic / neuroimaging	Small cohort; healthy participants; microbial composition/ENS function not directly linked to brain changes
Bifidobacterium longum NCC3001 (Pinto-Sanchez et al., 2017)	Microbiota-associated gut–brain signaling	Randomized, double-blind, placebo-controlled trial in IBS; fMRI	*n* = 44 randomized (22/22; 38 completed)	Reduced depression scores and altered responses to emotional stimuli in several brain regions; no significant improvement in IBS symptoms	No direct ENS evidence; CNS neuronal endpoint	Human clinical + neuroimaging	Small pilot study; changes in brain activity cannot be attributed specifically to ENS signaling
FMT in Parkinson’s disease (Scheperjans et al., 2024)	Gut microbiota modification	Double-blind, randomized, placebo-controlled trial; 12-month follow-up	48 randomized; 45 ITT	Sustained microbiota changes but no clinically meaningful improvement in PD symptoms vs. placebo	No direct ENS evidence; neurological clinical endpoints	Human randomized clinical trial	No neuronal/ENS activity measured; microbiota changes did not translate into clinical benefit

ASC, apoptosis–associated speck–like protein containing a CARD; CNS, central nervous system; ENS, enteric nervous system; FMPP, fermented milk product with probiotics; fMRI, functional magnetic resonance imaging; FMT, fecal microbiota transplantation; GI, gastrointestinal; IBS, irritable bowel syndrome; IL, interleukin; ITT, intention-to-treat; Panx1, pannexin-1; PD, Parkinson’s disease; P2X7R, P2X7 receptor; SCFAs, short-chain fatty acids; VNS, vagus nerve stimulation.

Experimental and clinical studies suggest that microbiota-targeted interventions can influence intestinal physiology, visceral sensitivity, and neuroimmune signaling. In patients with irritable bowel syndrome, supplementation with Bifidobacterium infantis improved several gastrointestinal symptoms and normalized an altered IL-10/IL-12 ratio, indicating clinical and immunomodulatory effects without directly demonstrating ENS modulation (O’Mahony et al., 2005). In experimental models, microbiota-dependent modulation of ENS maturation and intestinal motility has been demonstrated in germ-free and conventionalized mice and involves host serotonergic signaling through 5-HT_4_ receptors (De Vadder et al., 2018).

Clinical evidence directly linking microbiota-targeted interventions to neuronal activity remains limited. A small number of randomized studies have combined microbiota-directed interventions with central neuronal or neurological outcomes. In healthy women, consumption of a fermented milk product containing probiotics altered activity and connectivity within brain networks involved in emotional and viscerosensory processing (Tillisch et al., 2013). Similarly, in patients with IBS, Bifidobacterium longum NCC3001 reduced depression scores and altered brain responses to emotional stimuli assessed by functional magnetic resonance imaging, although IBS symptoms were not significantly improved (Pinto-Sanchez et al., 2017). In Parkinson’s disease, a randomized placebo-controlled trial demonstrated sustained modification of gut microbial composition following FMT but no clinically meaningful improvement in neurological symptoms compared with placebo (Scheperjans et al., 2024). Importantly, while these studies provide clinical evidence that microbiota-targeted interventions can be associated with central or neurological outcomes, none directly assessed enteric neuronal activity or ENS remodeling. Thus, direct clinical evidence linking therapeutic microbiota modulation to restoration of ENS function remains lacking.

Vagus nerve stimulation represents a mechanistically distinct approach that primarily targets neuroimmune pathways rather than intrinsic enteric neural circuits. Small prospective clinical studies in patients with Crohn’s disease have reported reductions in clinical disease activity and inflammatory markers, with evidence of endoscopic improvement in some patients (Bonaz et al., 2016; Sinniger et al., 2020; D’Haens et al., 2023). However, these studies were small and predominantly uncontrolled, and none directly assessed ENS function or remodeling. Experimental studies further demonstrate that ENS-specific pathways can represent potential therapeutic targets. In experimental colitis, inhibition of neuronal P2 * 7 receptor–pannexin-1 signaling prevented enteric neuronal death and associated alterations in gastrointestinal motility, providing mechanistic evidence that targeting enteric neuronal pathways can modify disease-associated neuroenteric remodeling (Gulbransen et al., 2012). However, such ENS-directed approaches remain predominantly preclinical. Overall, most currently available therapeutic approaches act upstream or systemically and do not directly target enteric neuronal circuits, ENS plasticity, or microbiota-dependent neuroepithelial signaling mechanisms. Consequently, the specific contribution of ENS remodeling to therapeutic efficacy often remains difficult to dissect. Moreover, substantial interindividual variability in microbiota composition, disease phenotype, and neuroimmune responsiveness further complicates the development of targeted microbiota–ENS therapies. These limitations underscore the need for mechanistically informed therapeutic strategies capable of selectively modulating ENS-associated signaling pathways and adaptive neuroenteric plasticity.

## 4 Discussion

### 4.1 Key limitations

Despite growing interest in the microbiota–ENS axis, several critical limitations continue to hinder translational progress. One major challenge is the lack of ENS-specific functional readouts and standardized biomarkers capable of reliably assessing enteric neuronal activity, neuroepithelial communication, and ENS plasticity *in vivo*. Although *ex vivo* electrophysiological recordings, calcium imaging, and neurochemical analyses have substantially advanced experimental ENS research, comparable approaches for longitudinal investigation of human ENS function remain limited (Boesmans et al., 2013; Spencer and Hu, 2020; Elfers et al., 2025b; Fung et al., 2025). In addition, substantial differences between experimental animal models and human disease conditions complicate mechanistic interpretation and translational applicability. Germ-free and antibiotic-depletion models have provided fundamental insights into microbiota-dependent ENS regulation and neuronal plasticity (De Vadder et al., 2018; Vicentini et al., 2021), yet they only partially reproduce the complexity, chronicity, and interindividual variability of human gastrointestinal and gut–brain disorders. Moreover, many experimental studies focus on acute perturbations, whereas maladaptive ENS remodeling likely develops progressively over prolonged periods of chronic inflammation, dysbiosis, and environmental exposure.

Another major limitation is the insufficient integration of microbiota-derived metabolomic data with functional neuronal, epithelial, immune, and clinical readouts. While alterations in microbial composition, metabolite availability, barrier function, and enteric neuronal signaling have individually been described in experimental and clinical studies (Yano et al., 2015; Obata et al., 2020; Elfers et al., 2024), these systems are still frequently investigated in isolation despite increasing evidence for dynamic bidirectional interactions within the microbiota–epithelial–immune–ENS axis. Consequently, the precise molecular mechanisms linking dysbiosis-associated metabolite alterations to long-term ENS remodeling and disturbed gut–brain signaling remain incompletely understood.

Collectively, these challenges restrict mechanistic interpretation of therapeutic outcomes and slow the development of targeted neuroenteric therapies. Future progress will therefore require integrative and longitudinal approaches combining microbiota profiling, metabolomics, neurophysiology, epithelial biology, and computational modeling to better characterize adaptive and maladaptive ENS plasticity in health and disease.

### 4.2 Future directions and emerging methodologies

Future progress in microbiota–ENS research will depend on integrative and mechanistically oriented approaches capable of linking microbial composition, metabolite production, epithelial physiology, immune activation, and ENS function within unified analytical frameworks. Such multidimensional strategies are particularly important because microbiota-dependent ENS regulation emerges from dynamic interactions among neuronal, epithelial, immune, and microbial systems rather than isolated signaling pathways.

In this context, non-invasive microbiome and metabolomic profiling strategies combined with functional analysis of fecal or luminal supernatants may provide clinically accessible insights into microbiota-derived neuroactive signaling and its effects on enteric neuronal activity (Yano et al., 2015; Elfers et al., 2024). Parallel advances in electrophysiology, calcium imaging, and human ENS neuroimaging increasingly enable direct functional characterization of enteric neuronal circuits and neuron–glia interactions under physiological and pathological conditions (Boesmans et al., 2013; Elfers et al., 2025b).

Emerging human-relevant model systems, including intestinal and enteric organoids, further offer promising opportunities to investigate microbiota–epithelial–neuronal interactions while reducing species-specific translational limitations. In parallel, single-cell transcriptomic approaches are beginning to reveal ENS-specific neuronal and glial signaling profiles with unprecedented cellular resolution (Obata et al., 2020). Finally, integration of microbiome, metabolomic, neuronal, and clinical datasets through computational and in silico modeling approaches may facilitate predictive disease stratification, biomarker discovery, and personalized therapeutic targeting of the microbiota–ENS axis, thereby improving clinical management through faster diagnosis, enhanced patient stratification, and the development of individualized therapeutic strategies.

Collectively, such multidisciplinary approaches may help bridge current mechanistic gaps and improve understanding of adaptive and maladaptive neuroenteric plasticity in gastrointestinal and gut–brain axis disorders.

## 5 Summary and outlook

The enteric nervous system is increasingly recognized as a dynamic and highly plastic integrative network positioned at the interface between the intestinal microbiota, epithelial barrier, immune system, and gut–brain axis. Rather than functioning solely as an autonomous regulator of intestinal motility and secretion, the ENS continuously senses and integrates microbial, metabolic, immune, and environmental signals to coordinate intestinal homeostasis and adaptive physiological responses.

Accumulating evidence indicates that chronic dysbiosis, inflammation, and environmental perturbations can disrupt this adaptive plasticity and promote maladaptive neuroenteric remodeling characterized by altered neuronal excitability, disturbed neuroimmune communication, and impaired gut–brain signaling. However, many of the molecular and cellular mechanisms linking microbiota-derived signals to long-term ENS remodeling remain incompletely understood.

Future progress in the field will depend on integrative and mechanistically oriented approaches capable of combining microbiome analysis, metabolomics, neurophysiology, epithelial biology, and computational modeling within clinically relevant systems. A deeper understanding of microbiota–ENS communication may ultimately enable the development of precision-targeted therapeutic strategies aimed at restoring adaptive neuroenteric signaling in gastrointestinal and gut–brain axis disorders.
